# Editorial: The impact of extreme weather events on public health

**DOI:** 10.3389/fpubh.2025.1645681

**Published:** 2025-07-25

**Authors:** Michael Tong

**Affiliations:** National Centre for Epidemiology and Population Health, Australian National University, Canberra, ACT, Australia

**Keywords:** climate change, extreme weather events, environmental health, public health, health risk assessment, climate change adaptation, heatwave, flood

Anthropogenic climate change has intensified the frequency, severity, and complexity of extreme weather events worldwide, as underscored by the Intergovernmental Panel on Climate Change's Sixth Assessment Report (1). Heatwaves, droughts, wildfires, cyclones, and heavy precipitation pose escalating threats to public health, with consequences observed across multiple domains. From the record-breaking heatwaves in Europe to the devastating droughts in Africa and destructive wildfires in America (2–4), the public health implications are both immediate and profound. These events drive a wide range of adverse health outcomes, including heat-related illnesses, exacerbation of chronic diseases, physical injuries, significant mental health burdens, and excess mortality. However, these health impacts of extreme weather events are heterogeneous and shaped by factors such as population vulnerability, socio-economic conditions, and regional climatic variability.

This Research Topic brings together nine articles that collectively advance our understanding of these complex dynamics, integrating epidemiological, mechanistic, and intervention-focused research, and disaster communication case study to inform future research, health policies, and public health practices.

A prominent theme across the collection is the public health impact of climate change and extreme heat. Chen S. et al. employed a novel application of bivariate frequency analysis using copula functions, that offers a refined approach to quantify heatwave characteristics and associated mortality risks in urban China. This methodological innovation offers accurate forecasting and adaptive planning in cities increasingly vulnerable to extreme heat. Further, contributions extend beyond epidemiology to illuminate the biological mechanisms underpinning the health impacts. Wen et al.'s longitudinal multi-omics profiling study showed intricate molecular responses to acute heat exposure, identifying novel biomarkers predictive of cardiopulmonary function changes. Such mechanistic insights deepen our understanding of the physiological stress imposed by extreme heat and may guide the development of clinical interventions. Complementing these studies, Ning et al. investigated the synergistic effects of cold spells and particulate matter pollution on mortality in a high-altitude setting, highlighting the compounded health risks from multiple environmental stressors. The study underscored the necessity of integrating meteorological and pollution exposures to fully capture public health risks.

Mental health impacts of climate change related extreme weather events are also prominently featured. Akram and Mushtaq's mini-review shed light on the often-overlooked mental health consequences of displacement due to flooding, emphasizing the importance of incorporating psychological support into disaster response frameworks. Moreover, Chen D-D et al.'s systematic review and meta-analysis of climate change and suicide revealed high temperatures and air pollution increased suicide attempts, suicide deaths, anxiety and self-harm, emphasizing the need for targeted public health strategies.

Another contribution comes from Zou and Lv, who analyzed social media data to track public discussion of disaster risk and emergency knowledge across the different phases of Typhoon Yagi in China during the typhoon warning, occurrence and recovery period. This highlights the co-evolutionary nature of disaster risk diffusion and emergency knowledge dissemination, providing a case study for future responses to extreme weather events on disaster risk management and emergency knowledge dissemination.

Studies in this collection underscore the disproportionate risks faced by vulnerable populations. Sankar et al. describe a protocol for evaluating the effectiveness of heat stress interventions among outdoor workers, a group at the frontline of climate exposure. Addressing occupational heat risk is a critical priority in low- and middle-income settings where adaptation capacity is limited.

In addition, Thongsak et al. link long-term exposure to air pollution from crop burning and forest fires to increased liver cancer mortality, adding to the growing evidence on chronic disease burdens exacerbated by environmental degradation in the context of climate change, particularly from the forest fires or wildfires due to climate change. Their findings reinforce the need for air quality control as part of comprehensive climate and health strategies.

Collectively, these studies underscore the multifaceted nature of extreme weather impacts on health, spanning acute and chronic outcomes, physical and mental health. They also highlight the urgent need for interdisciplinary approaches that combine advanced data analytics, molecular biology, social science, and occupational health to develop effective adaptation and mitigation strategies.

Importantly, this body of work points toward practical avenues for public health interventions. These include the development of heat stress management protocols tailored to vulnerable workers, incorporation of mental health services into disaster response, deployment of real-time risk communication strategies, and application of novel biomarkers for health monitoring during extreme heat exposure. As climate change continues to intensify the frequency and severity of extreme weather events, such evidence-based strategies will be essential to protect health and promote resilience.

This Research Topic serves as a timely resource, synthesizing cutting-edge research that bridges gaps in knowledge and offers practical insights for policymakers, public health professionals, clinical practitioners, and researchers. The integration of epidemiological data, mechanistic studies, intervention frameworks, disaster response and management offer a comprehensive lens to understand and address the public health challenges posed by extreme weather events. Continued investment in interdisciplinary research and cross-sector collaboration will be essential to enhance resilience and protect vulnerable populations due to extreme weather events in the face of climate change.
